# Alumni Association of the Dental Department of the University of Maryland

**Published:** 1883-04

**Authors:** 


The exercises closed with the benediction, pronounced by Rev. Dr. Hodges.

The Alumni of the Dental Department of the University have elected the following officers: President, Dr. R. Arthur Hungerford, of Maryland; Vice Presidents—R. D. Dodson, of Pennsylvania; Dr. G. I. Grempler, of Maryland; Dr. J. F. Garrett, of North Carolina; Secretary and Treasurer, Dr. A. Lee Penuel, of Maryland.

The fifth annual reunion and banquet of the alumni association of the University took place last evening at the Eutaw House. Dr. N. S. Lincoln, of the class of 1859, and now located at Washington, D. C., delivered an address on the advantages of a classical education as a preliminary to medical study. After the reunion of the alumni of the University from different parts of the country, members of the graduating classes of the present year, and a number of invited guests, sat down to a banquet. On the removal of the cloth several toasts were given and responded to.

The success of the first Session of this Dental Department, which ended on the 15th of March last, is unprecedented in the history of any dental institution. The matriculates for the Session numbered sixty-six, all of whom, without a single exception were dental students and not matriculates of the Medical Department, or of any other medical college. Of the graduates eighteen had attended their first session at other Dental Colleges.

